# The Insane and the Law

**Published:** 1911-10-07

**Authors:** 


### SPECIAL ARTICLES.

### The Insane and the Law.

### HOW ORGANISED TREATMENT OF THE INSANE BEGAN.

The most mysterious and fascinating subject of research through all ages has been the human mind itself, and until recent times its pathological manifestations have been generally considered to be due to supernatural agency. That mental alienation existed and was recognised in very early times is proved by the fact that the symptoms of senile dementia are described in an Egyptian papyrus which dates about 4500 B.C. It seems to have occurred in all ages and in every country, and though the superstitious and ignorant have generally regarded the insane as being possessed by some supernatural power, good or evil, yet it appears probable that the thinking few have always recognised it as unwholesome. This superstition of demoniacal possession is still to be found, even amongst educated people, though epilepsy—the sacred malady of the ancients—has for generations been regarded as a pathological stigma; and the writer has had under his care a lady suffering from acute mania from whom a clergyman, apparently sane, had attempted with Bible and crucifix to exorcise the evil spirit which, according to him, had entered into her and was the cause of her mania. Such cases as this are, of course, rare, yet it is curious that there should be so much ignorance of the elements of mental pathology amongst the laity in an age when to label any book " a psychological study " seems to ensure its being read.

#### Classification of the Insane.

One can judge what probably happened to the insane in early times from what becomes of them amongst savage and uncivilised tribes. For this purpose insanities may be roughly classified into three divisions: (1) Congenital mental deficiency—idiots and imbeciles; (2) Insanities occurring in adults—in which a marked change takes place in the hitherto normal mental state of the individual; (3) Senile dementia—the result of wear and tear of the nervous system showing itself in advancing years. Amongst uncivilised peoples the lowest grade of idiots and imbeciles, if not killed in early infancy, die of neglect; higher grades, who are able to provide for themselves, survive until their cunning and bad temper are abruptly determined by a sane member of the community and a club. Cases of senile dementia, or second childhood, as the condition seems to have been called in Ancient Egypt, which are now carefully looked after, are reported to be killed in many tribes as soon as they become a burden. Most interest lies in the cases of insanity occurring in adults, and these would appear to die as a result of their disease, to be killed as a result of their delusions, or to be held sacred as possessed of supernatural powers and carefully tended. With advancing civilisation the slaying in cold blood ceased, but for the active amelioration of the condition little or nothing was attempted. Legislation as regarding the insane commenced in different countries when civilisation reached such a state that waste of property was recognised as an evil; and in the resulting laws the property and not the patient was the first consideration. Amongst the Romans there was apparently no segregation of the insane, but the law restricting freedom of action was more rigorous than our own. According to the law of the Twelve Tables, madmen and prodigals were placed under the care of specially appointed guardians, who managed their affairs. (The classing of the prodigal with the insane is interesting and shows wisdom). In England, the Statute de Prerogativa Regis in 1324 made the king the guardian of idiots and madmen, to administer their estates, a duty which had hitherto been in the hands of the overlord. Upon the Lord Chancellor, as keeper of the King's conscience and presiding officer of the Court of Chancery, the duties naturally devolved, and he has continued to fulfil them ever since, being at the present day the President of the Lunacy Commission.

#### Early Institutions.

Though legislation for dealing with the property of the insane began so early, it was not until the end of the eighteenth century that any law was passed dealing with the care of the patient. At that time there were at least three philanthropic institutions for dealing with the poor, and many private ones for dealing with the well-to-do patients. The philanthropic institutions alluded to were three of the registered hospitals of the present day: Bethlem Hospital, founded in 1247; the Bethel Hospital at Norwich, founded in 1711; and St. Luke's Hospital, founded in 1750. These dealt with acute cases who could not afford to pay the fees of the private houses, but did not take in parish poor; and such treatment as was then in vogue was carried out in them. The first Lunacy Act dealing with the welfare of the patient, passed, as stated above, at the end of the eighteenth century, was largely due to the public attention having been directed to the condition of the existing asylums by the insanity of King George III. Non-restraint treatment also began about this time with the opening of the York Retreat by Tuke, whilst the same work was being carried on in France by Pinel. Soon after this the first Poor Law asylums were opened, and improvements in the law were made. There was but little progress in scientific treatment, however, until the middle of last century, though interest in the subject continued to grow and its importance became more fully realised. The present Lunacy Act and its amendment, dating from 1890-91, is now badly in need of revision, which, though long since promised, does not seem likely to be accomplished for some time. Our asylums, the treatment carried out in them, and the utilisation of the vast field for scientific research that they afford, though immensely improved do not yet approach the ideal: nor does it seem likely that they will do so until the care of the insane is undertaken as a whole by the State instead of by numerous local authorities.

